# Pigmentary and photonic coloration mechanisms reveal taxonomic relationships of the Cattlehearts (Lepidoptera: Papilionidae: *Parides*)

**DOI:** 10.1186/s12862-014-0160-9

**Published:** 2014-07-27

**Authors:** Bodo D Wilts, Natasja IJbema, Doekele G Stavenga

**Affiliations:** 1 Computational Physics, Zernike Institute for Advanced Materials, University of Groningen, Nijenborgh 4, Groningen, NL-9747AG, The Netherlands; 2 Present address: Department of Physics, Cavendish Laboratories, University of Cambridge, 13 JJ Thomson Avenue, Cambridge, CB3 0HE, UK; 3 Present address: Accenture Nederland B.V, Gustav Mahlerplein 90, Amsterdam, NL-1082 MA, The Netherlands

**Keywords:** Iridescence, Morphology, Papilionidae, Papiliochrome, Phylogeny, Scattering, Wing traits

## Abstract

**Background:**

The colorful wing patterns of butterflies, a prime example of biodiversity, can change dramatically within closely related species. Wing pattern diversity is specifically present among papilionid butterflies. Whether a correlation between color and the evolution of these butterflies exists so far remained unsolved.

**Results:**

We here investigate the Cattlehearts, *Parides*, a small Neotropical genus of papilionid butterflies with 36 members, the wings of which are marked by distinctly colored patches. By applying various physical techniques, we investigate the coloration toolkit of the wing scales. The wing scales contain two different, wavelength-selective absorbing pigments, causing pigmentary colorations. Scale ridges with multilayered lamellae, lumen multilayers or gyroid photonic crystals in the scale lumen create structural colors that are variously combined with these pigmentary colors.

**Conclusions:**

The pigmentary and structural traits strongly correlate with the taxonomical distribution of *Parides* species. The experimental findings add crucial insight into the evolution of butterfly wing scales and show the importance of morphological parameter mapping for butterfly phylogenetics.

## Background

The wing coloration and patterning of a butterfly play important roles in its everyday survival. The coloration pattern can function as a concealment or disguise signal for protection (camouflage, crypsis) or for attracting potential mates (display) [1],[2]. Coloration furthermore is an important aspect in mimicry, i.e. the parallel evolution of species that have evolved optically similar traits. Indeed, color is an important element of species selection mechanisms as many species have evolved by means of diverging coloration [3]. Evolutionary mechanisms of coloration appeared independently in multiple phylogenetic places, contributing largely to the rich biodiversity of life present on earth today [4]. Butterflies are a prime example of biodiversity [5],[6]. Even though extensively investigated in the past, it remains unclear whether their phylogeny and wing color are correlated.

**Figure 1:**
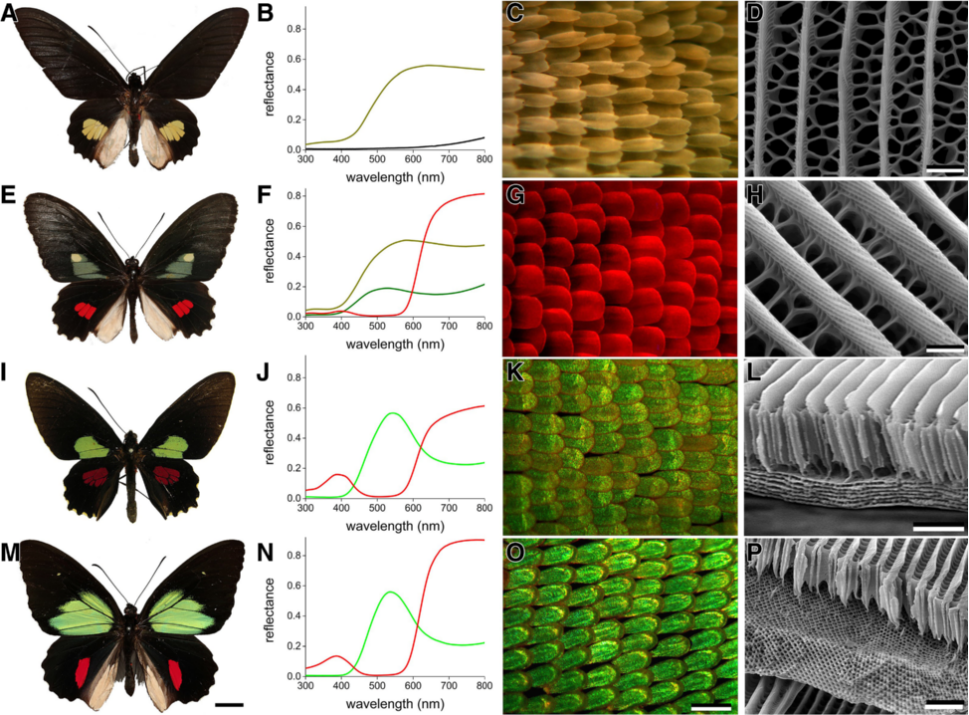
***Parides*species with differently structured and colored wing scales. A***Parides quadratus*. The dorsal hindwings have yellow colored patches, the rest of the butterfly is black. **B** Reflectance spectra of yellow and black wing areas. The color of the spectral curves corresponds to the scale color. **C** Light micrograph of the wing area with yellow scales. **D** SEM image of a single scale. **E***P. erithalion zeuxis*. The dorsal forewings have an olive-colored area with additionally a small yellow spot, and the hindwings have red spots. **F** Reflectance spectra of the olive-colored, yellow and red wing areas. **G** Micrograph of the wing area with red scales. **H**. SEM image of a red scale showing the ridge lamellae forming multilayers. **I***P. aeneas bolivar.* The dorsal forewings have green-colored areas, and the hindwings have burgundy spots. **J** Reflectance spectra of the green and red wing areas. **K** Micrograph of the green wing area. **L** SEM image of a cut green wing scale showing a multilayer structure in the scale lumen with a very elaborate upper lamina. **M***P. childrenae oedippus*. The dorsal forewings have large, brightly green-colored areas, and the hindwings have bright red spots. **N** Reflectance spectra of the green and red wing areas. **O** Micrograph of the green scales, which have a metallic appearance and small domains varying in color. **P** SEM image of a fractured scale, revealing a 3D-structure below the elaborate upper lamina. Scale bars: A,E,I,M - 1 cm; C,G,K,O - 100 µm; D,H,L,P - 1 µm.

Here, we investigate a possible color-phylogeny correlation in a small genus of papilionid butterflies, the Cattlehearts (*Parides*; Huebner, 1819), a Neotropical New World taxon with 36 members [7],[8]. The coloration of *Parides* butterflies is quite stunning and diverse (Figure 1). The wings are predominantly black, but distinct wing patches feature vividly colored scales that can strongly differ between species. The colors and arrangement of these brightly colored spots on the butterfly wings serve as an aposematic signal to birds [9]. Aposematism is an antipredatory strategy where a potential prey radiates its unpalatability by a signal that is readily understood by predators [10]. With their prominent color patterns, *Parides* butterflies are suitable for Müllerian mimicry rings. Their predators – especially birds – quickly learn to recognize these patterns and become averse to it [11].

Coloration is generally divided into two classes: pigmentary (chemical) coloration, due to wavelength-selective light absorption by pigments, and structural (physical) coloration, due to wavelength-selective light reflection resulting from incident light interacting with nanostructured matter [12]. Up to date, of the Cattlehearts only the coloration principles of the Emerald-Patched Cattleheart, *P. sesostris*, have been elucidated in extensive detail [13]–[20]. The wing scales of the green patches at the dorsal wings of *P. sesostris* contain a single-network, gyroid-type photonic crystal layer, which reflects blue-green light, depending on the angle of illumination. However, the scales also contain a violet-absorbing pigment that functions as a spectral filter, causing an angle-independent, vivid-green wing color [19].

The small size of the genus with its large diversity in wing coloration makes the *Parides* an attractive study group for investigating possible color-phylogeny relationships. By applying various physical techniques [21], we investigated the coloration mechanisms of the male members of the genus *Parides.* We thus identified a restricted set of pigments and photonic structures that together create the vivid colors of *Parides* butterflies. We discuss the results in view of the evolution of butterfly wing scales and the taxonomic relationships of coloration in phylogenetic trees.

## Results

### Appearance of the wings and wing scale morphology

The dorsal wings of *Parides* butterflies feature brightly colored patches in a generally jet-black frame [22],[23]. Figure 1 presents an overview of the wing patterns of a few typical *Parides* species (Figure 1A,E,I,M). To investigate the nature of the variously colored wing areas, we measured reflectance spectra with a bifurcated probe (Figure 1B,F,J,N), and we made light micrographs of the scales in the colored wing areas (Figure 1C,G,K,O). We further performed scanning electron microscopy (SEM) to reveal the ultrastructure of the scales (Figure 1D,H,L,P). 

The dorsal wings of *P. quadratus* (Figure 1A) are largely black with small yellow patches in the hindwings. As expected for its yellow color, the reflectance spectrum of the wing patches is low in the ultraviolet and high in the long-wavelength range (Figure 1B). The reflectance of the black wing areas is minimal in the ultraviolet and visible wavelength ranges but becomes noticeable in the far-red, which is characteristic for high concentrations of melanin (Figure 1B). The yellow scales are densely stacked in a regular lattice (Figure 1C). Scanning electron microscopy shows that the scales are built according to the basic bauplan of papilionid butterfly wing scales [23],[24]; the upper surface features parallel ridges, with in between randomly arranged cross-ribs, which form large windows (Figure 1D).

**Figure 2:**
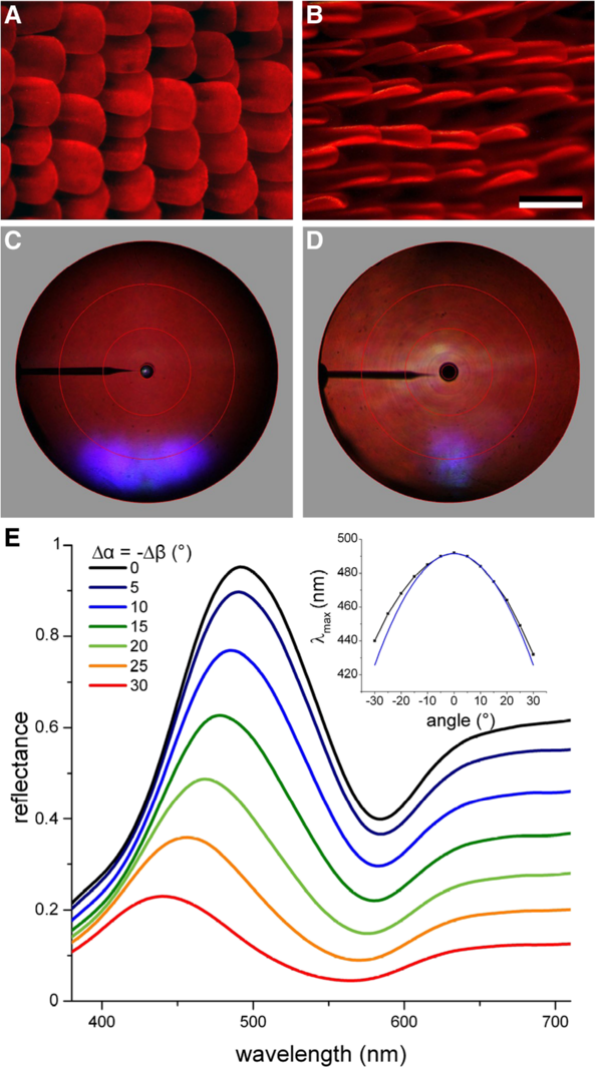
**Red scales with blue iridescence of*P. anchises*and*P. lysander*. A**, **B** Whereas the scales of *P. anchises* are about flat, those of *P. lysander* are highly curved. **C**, **D** Scatterograms of the two differently-shaped scales. **E** Angle-dependent reflectance spectra of a wing piece of *P. anchises* measured with a two-fiber goniometric setup. The illumination angle (Δ*α*) and detection angle (Δ*β*) were changed in steps of 5 degrees in opposite directions and symmetrically with respect to the angle of peak reflectance, which was at ~60° with respect to the normal to the wing plane. Inset: reflectance peak wavelength as a function of light incidence fitted with the interference condition for multilayers.

The dorsal wings of *P. erithalion zeuxis* (Figure 1E) display various colors: olive, yellow, and red. The reflectance spectra vary accordingly (Figure 1F) and indicate different pigments as well as scale structures. The wing patches of the hindwings are covered by densely stacked red scales (Figure 1G) The anatomy of the wing scales is similar to that shown in Figure 1D, but the ridges are taller due to a large stack of overlapping ridge lamellae, which together create a slender, highly-tilted multilayer (Figure 1H). The multilayered scales show, with normal illumination of the wing, a strong blue-iridescence in an oblique direction (see below, Figure 2).

The dorsal forewings of *P. aeneas bolivar* (Figure 1I) have green patches and the hindwings red patches. Their reflectance spectra contrast strongly (Figure 1J). The quite pronounced green reflectance indicates a structural origin, which is also suggested by the gleam of the wing scales (Figure 1K). Indeed, scanning electron microscopy revealed elaborately structured scales (Figure 1L). The scale lumen consists of a large multilayer, and the scale’s upper lamina has tall ridges connected with a thick layer of cross-ribs, quite in contrast with the basic pattern of moderate ridges and thin cross-ribs (Figure 1D).

Of the species shown in Figure 1, *P. childrenae oedippus* (Figure 1M) has the most striking color pattern. The reflectance spectra of the green and red patches are similar to those of *P. aeneas*, suggesting similar coloration mechanisms. However, with epi-illumination light microscopy the green scales have a rather different optical appearance, especially a different gloss, indicating the presence of dissimilar photonic structures (Figure 1O). Scanning electron microscopy of a fractured scale revealed a 3D network-like structure in the scale lumen (Figure 1P), which was previously demonstrated to be a gyroid-type photonic crystal [15],[17]–[20].

### Pigmentary coloration

**Figure 3:**
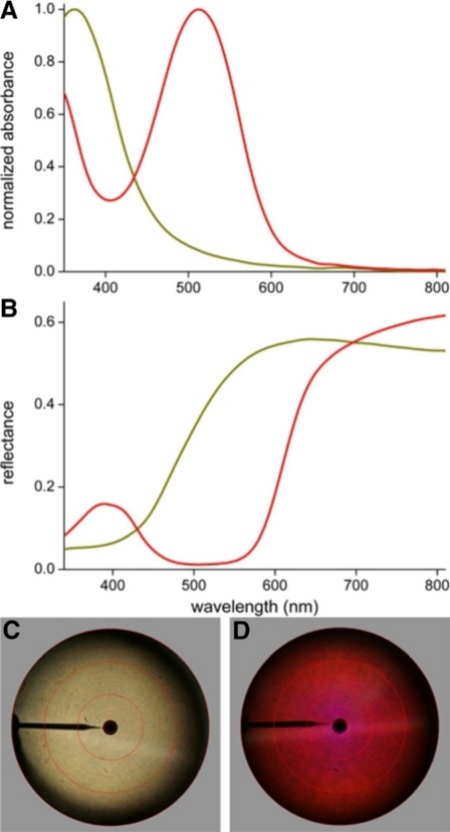
**Absorbance and reflectance spectra and scatterograms of single yellow and red wing scales of*P. aeanas bolivar*and*P. sesostris*. A** Absorbance spectra calculated from transmittance spectra measured of single wing scales immersed in refractive index matching fluid. **B** Reflectance spectra measured with a microspectrometer. **C**, **D** Scatterograms showing wide-angled, diffuse scattering of a single yellow scale of *P. aeanas bolivar***(C)** and a single red wing scale of *P. sesostris***(D)**. The about horizontal lines are due to diffractive scattering by the scale ridges, which were oriented about vertically. The red circles indicate scattering angles of 5°, 30°, 60° and 90°, and the black bar at 9 o’clock is due to the glass micropipette holding the scale.

The reflectance spectra of the yellow and red scale patches (Figure 1B,F,J,N) indicate the presence of two different, short-wavelength absorbing pigments. To identify the pigments, we immersed single scales in refractive index matching fluid (*n* = 1.55) and measured transmittance spectra with a microspectrophotometer (MSP) (Figure 3A). The differently colored scales yielded two classes of absorbance spectra (Figure 3A). The yellow, olive and green colored scales all contained a pigment with peak wavelength ~390 nm and all red scales contained a pigment with peak wavelength ~520 nm.

We also measured with the MSP reflectance spectra of small scale areas (diameter ~15 μm) of isolated single scales (Figure 3B). These spectra can be readily understood from the absorbance spectra of Figure 3A, because the pigments selectively absorb incident light in restricted wavelength ranges and thus the pigments reduce the amount of backscattered light. This is further illustrated by the scatterograms of a yellow and red scale. Illumination of a small scale area with a narrow-aperture white light beam results in both cases in a spatially wide angled, diffuse light distribution that virtually fills the whole hemisphere (Figure 3C,D).

### Structural coloration mechanisms

Additionally to pigmentary coloration, three distinct forms of structural coloration occur in the wing scales of *Parides* butterflies: ridge multilayers (Figure 1H), lumen multilayers (Figure 1L) and gyroid-type photonic crystals (Figure 1P). We have investigated the consequences of these scale structures for light reflection with scatterometry and by measuring reflectance spectra as a function of illumination angle.

#### Ridge multilayers

Scanning electron microscopy demonstrates that the red scales of *P. anchises* (Figure 2A) and *P. lysander* (Figure 2B) both have ridges with tilted lamellar stacks, like those of *P. erithalion zeuxis* (Figure 1G). The red scales of *P. anchises* are about flat, but those of *P. lysander* are highly curved in a gutter-like shape. The scatterograms of the two scale types, obtained with a narrow-aperture white light beam, show a wide-field distribution of red light (Figure 2C,D; see Figure 3D), but in addition they feature highly-directional blue reflections. The highly directional reflection emerges from the strongly tilted multilayers created by stacked lamellae in the scale ridges. Scale ridges with similar stacked lamellae exists in other papilionids [25],[26], and are even more pronounced in *Morpho*[12] and pierid [27],[28] butterflies. The scatterograms, which are obtained with normal illumination, show at scattering angles of ~60-70° that the stacks act as interference reflectors with tilt angle of 30-35° with respect to the wing plane. The ridges are slender and they therefore diffract incident light in a plane perpendicular to the ridges (see also [29]), but the angular spread of the blue reflection of the scales of *P. anchises* (Figure 1C) is much wider than that of *P. lysander* (Figure 1D). This is obviously related to the different shape of the scales, being flat and curved, respectively.

We further investigated the angle-dependence of the blue reflection using the ARM setup, consisting of two optical fibers that can be rotated around the sample (see *Methods*). We first aligned the illumination and detection fiber and determined the angle where the reflectance was maximal. This was called the normal position. We then measured reflectance spectra as a function of the angle of light incidence relative to the normal position, Δ*α*, whilst the detection angle, Δ*β*, was set at the mirror position: Δ*β* = −Δ*α* (Figure 2E). When increasing the angle of incidence, the reflectance shifted hypsochromically, i.e. towards shorter wavelengths, as expected for a multilayer [30],[31]. We compared the angle-dependent shift in peak wavelength derived from Figure 2E, plotted in the inset, with values following from the interference condition for reflecting multilayers (see Methods) by using data for the thickness of the air (*d*_a_) and cuticle layers (*d*_c_) estimated by scanning electron microscopy: *d*_a_ ≈ 82 nm and *d*_c_ ≈ 105 nm (blue line). With refractive indices *n*_a_ = 1 and *n*_c_ = 1.56 a reasonable correspondence was obtained (Figure 2E, inset). The reflectance amplitude decreased with increasing angle, which is presumably due to the small size of the multilayers.

#### Lumen multilayers

**Figure 4:**
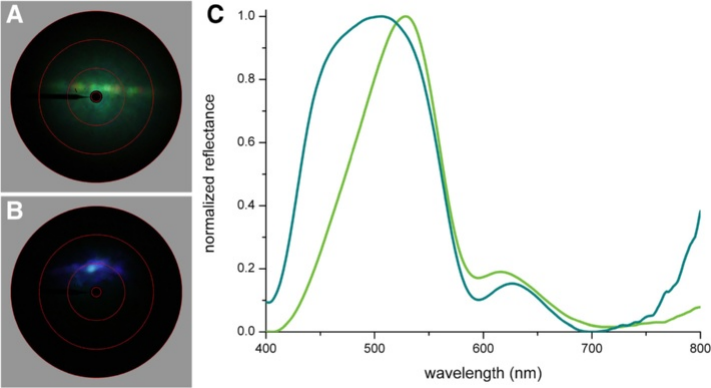
**Scatterograms and reflectance spectra of a green cover scale of*P. aeneas*illuminated with a narrow-aperture, white-light beam. A** Scatterogram of the upper side of the scale showing a diffraction pattern upon a diffuse background. **B** Scatterogram of the underside of the scale showing a strongly directional blue reflection. **C** Reflectance spectra of the upper (green curve) and under (olive curve) side of the scale.

The scales in the green forewing patches of *P. aeneas* contain a multilayer in the scale lumen, causing a bright-green gleam (Figure 1K,L). The interference reflection by the multilayer can be eliminated by immersing the scales in refractive index fluid. Transmittance measurements on immersed green scales demonstrated the presence of the violet-absorbing pigment (Figure 3B). The scales’ upper lamina is very elaborate (Figure 1L) and thus might contain a substantial fraction of the pigment (see also Additional file 1: Figure S1). Because a high pigment concentration may be expected to severely change the spatial and spectral reflectance characteristics of the wing scales [32], we investigated the reflection properties of both sides of the scales that is, of the upper, abwing side as well as the lower, adwing side, using our scatterometer and the MSP (Figure 4).

The scatterogram of the upperside shows a rather wide-field green scattering plus a clear indication of diffraction by the parallel array of ridges (Figure 4A). The scatterogram of the scale underside shows blue scattered light in a restricted spatial field (Figure 4B). The latter must represent the direct multilayer reflection. The slight spatial spread can be immediately understood from SEM observations that the scale underside is only approximately (but not perfectly) flat.

Reflectance measurements with the MSP yielded a narrow-band, green-peaking spectrum for the upperside and a much broader and more symmetrical blue-green-peaking spectrum for the underside (Figure 4C). Considering the scale’s anatomy (Figure 1L), the obvious interpretation of the difference in the reflectance spectra is that the spectrum of the underside is virtually fully determined by the multilayer reflector in the scale lumen, whilst the spectrum measured from the upperside represents the multilayer’s reflectance spectrum filtered by the violet-absorbing pigment.

**Figure 5:**
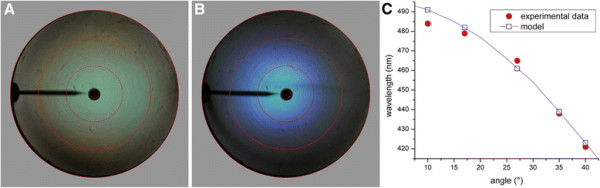
**Scatterograms and reflectance spectra of a single green scale of*Parides aeneas*illuminated with a wide-aperture, white-light beam. A**, **B** Scatterograms of the upperside and the underside of the scale with unpolarized light. **C** Reflectance peak wavelengths, derived from reflectance measurements, compared to peak wavelengths calculated with the interference condition of classical multilayer theory.

To further investigate the angle-dependence of the reflection and scattering by the green scales of *P. aeneas*, we performed scatterometry applying wide-aperture illumination. The scatterogram of the upperside then shows a virtually uniform green color diffusely spread throughout the hemisphere above the scale (Figure 5A), while the scatterogram of the scale’s underside is blue with hue depending on the spatial direction (Figure 5B). With increasing scattering angle, the color of the scattered light is blue-shifted, i.e. towards shorter wavelengths. Angle-dependent reflectance spectra from the underside measured with the scatterometer confirm the blue-shift, with a shift in peak wavelength from ~ 485 nm to 430 nm when the angle of incident is increased from 10 to 40° (Figure 5C, red dots).

The multilayer reflectance properties can be modeled when the layer thicknesses, the refractive indices and the number of layers are known. From SEM images we estimated the layer thickness as ~80 nm and the number of cuticle layers to be 7. The individual layers appeared to be not entirely uniform. The chitin layers are separated by air layers, but they are connected by tiny pillars, the trabeculae, raising the effective refractive index of the air layers to >1. Furthermore, the chitin layers are not smooth, but locally punctured with air holes, which reduces the effective refractive index of the cuticle layers to <1.56. Using refractive index values *n*_a_ = 1.09 and *n*_c_ = 1.45 (i.e. a cuticle filling fraction of 0.8), we could well reproduce the measured dependence of the reflectance peak wavelength on the angle of light incidence (Figure 5C and Additional file 1: Figure S2).

#### Gyroid-type photonic crystals

The striking green wing patches of e.g. *P. childrenae* and *P. sesostris* have scales with a 3D photonic crystal in the scale lumen with in the upper lamina a honeycomb-structured layer containing the violet-absorbing pigment (Figure 1L; see also [19]). The photonic crystal layer consists of a number of differently-oriented gyroid interference reflectors, reflecting predominantly in the blue-green wavelength range [17]–[20]. The broader reflectance spectrum of the gyroid reflector is narrowed to the observed vivid green due to the violet-absorbing pigment in the honeycomb layer [19], in a way that is surprisingly similar to that described above for the pigmented scales with a multilayer in the lumen.

### Color-phylogeny correlation

The different coloration mechanisms present in the Cattlehearts can be related to their phylogeny. The taxonomy of the family Papilionidae is generally well investigated, but only recently the genus *Parides* has been categorized by molecular techniques on the species-level with a limited number of species (18 out of 36 species). Phylogenetic information on the species-level for *Parides* butterflies has been published by Silva-Brandao et al. [7] based on DNA barcoding, and later slightly adjusted by Condamine et al. [4]. Further, Möhn et al. [33] classified the Cattlehearts into four major lineages depending on DNA results, habitat, external characteristics and color appearance.

**Figure 6:**
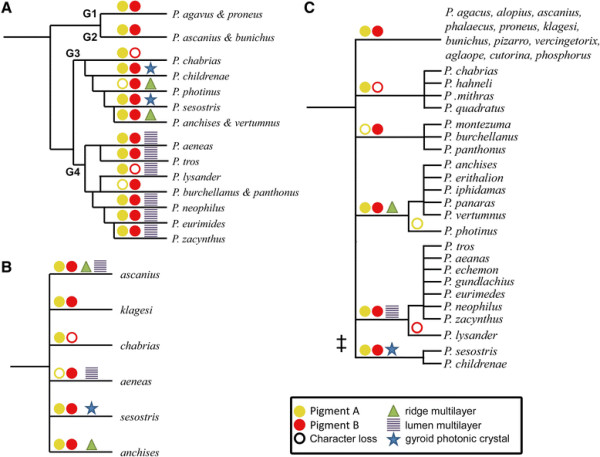
**Color-phylogeny correlation.** Morphological parameters and pigments mapped to phylogenetic trees on the species-level of Condamine et al. [4]**(A)** and by Möhn et al. [33]**(B)**; see also Additional file 1 and Additional file 2: Table S1. The yellow and red circles indicate loss of the yellow or red pigment. In **A**, G1-G4 indicates the group classification according to Silva-Brandao et al. [8]. **C** Phylogenetic tree that best resembles measured morphological parameters and pigment distribution with least instances of innovations. The double dagger indicates a suggested change in the developmental conditions of the wing scales resulting in a change from perforated multilayers to gyroid photonic crystals in the scale lumen (see text).

We used these two classifications to map the investigated pigmentary and structural parameters onto the phylogenetic trees. Additional file 2: Table S1 lists the presence of pigments (yellow or red) and the scale structures (multilayers in the ridges and/or lumen, or gyroids) and their location on the wing (fore- and/or hindwing) for a large number of Cattlehearts. The results are summarized in Figure 6. Virtually all species possess both pigments, but a few species have only one pigment (e.g. the yellow pigment is only present in some butterflies of the *chabrias* group and the red pigment in some butterflies of the *aeneas* group of species, Figure 6, see also Additional file 2: Table S1). This strongly suggests that both pigment types are not unique for the Cattlehearts. Indeed, the wing scales of butterfly genera closely related to *Parides*[4], e.g. *Troides* and *Ornithopthera*, contain the same pigments, which suggests that the pigments are ancestral to all these genera.

For the structural parameters, however, the mapping shows significant differences. The classification by Condamine et al. [4], the most finely sampled phylogeny to date (Figure 6A), divides the Cattlehearts in four groups. The butterflies in groups 1 and 2 do not have a special photonic structure, but those of group 3 have either a ridge multilayer or a gyroid photonic crystal. Most of the group 4 butterflies have a multilayer in the scale lumen. The phylogeny suggests multiple independent occurrences and losses of structural characters. This is specifically obvious in the occurrence (and loss) of the ridge multilayer structures and the gyroid-type photonic crystals, which in the current phylogeny appear to have evolved and got lost multiple times within group 3. This is a highly unlikely scenario, especially when considering the (potential) close relationship in the wing scale development of perforated multilayer structures and gyroids photonic crystals in the scale lumen (see below).

The classification by Möhn et al. [33] is rather coarsely sampled (Figure 6B). The structural parameters mostly occur in one specific group only, except for the *ascanius* species-group where both ridge and lumen multilayers occur. This supports a single evolutionary occurrence of these structures, much in contrast to the classification of Figure 6A. Especially in comparison with the finer sampled tree of Figure 6A, it seems likely that there has been a unique development of the trait rather than multiple independent occurrences. Thus, a species-level tree is necessary to draw specific conclusions on phylogeny.

Figure 6C presents an alternative phylogeny, based on the pigments and the morphological characters we identified. Albeit not being based on maximum parsimony or bootstrap methods, the tree minimizes the individual evolution of identical parameters. It will be interesting to investigate which result a combined phylogenetic analysis of morphological parameters and DNA barcoding would deliver [34].

## Discussion

### Coloration mechanisms in the Cattlehearts

Various coloration mechanisms are realized in the wing scales of *Parides* butterflies. Four different cases, each with a unique nanostructure, can be identified: i) the basic bauplan of papilionid butterflies, with a flat lower lamina and a structured upper lamina with ridges and irregular cross-ribs [23]; ii) tilted ridge lamellar stacks acting as multilayer reflectors, similar to those observed in *Morpho* and *Pieris* butterflies [12],[14],[28]; iii) perforated multilayer stacks in the scale lumen similar to those in lycaenid butterflies [35],[36], and iv) gyroid photonic crystal layers – as also observed in lycaenids [15],[18],[37].

Pigments in the scales affect the scales’ reflectance spectra. The ubiquitous pigment melanin, which has an absorption spectrum extending well beyond the visible wavelength range, causes brown or black scales, depending on the pigment concentration [38]. We identified two wavelength-selective absorbing pigments embedded in the cover scales of the different Cattleheart butterflies (Figure 3). The yellow scales of *P. quadratus* and the green scales of several Cattlehearts contain an (ultra)violet-absorbing pigment, which is most likely the well-known papiliochrome II of other papilionids [19],[32],[39]. The red scales of several Cattlehearts contain a blue-green-absorbing pigment, which has not been specifically characterized but presumably is also a papiliochrome type pigment [40]. The pigments are located (at least partly) in the upper lamina (Additional file 1: Figure S1), which is profuse in those cases where the scale lumen contains a photonic structure, a multilayer or a 3D gyroid-type photonic crystal. Both photonic structures specifically reflect blue-green light, and in both cases the reflectance spectra are narrowed down to the green wavelength range due to spectral filtering by the pigment [19]. The upper lamina is irregular structured and thus removes the intrinsic directionality of the reflected light and makes the color mostly angle-independent (Figure 5).

We found that the scales in some Cattlehearts are distinctly curved (Figure 2). This suggests that scale curvature also tunes the directionality of the reflected light (see also [41]). Furthermore, the jet-black framing, due to heavily melanized scales, creates a contrastful wing pattern, strongly increasing the visibility of the colored wing patches, for instance for potential predators or mates.

### Wing scale development

Closely related *Parides* species can have scales with in the lumen either perforated multilayer structures (*P. aeanas* and others) or gyroid photonic crystals (*P. childrenae* and *P. sesostris*, see Additional file 2: Table S1 and Figures 4, and 6). From a developmental point-of-view, it is rather difficult to assess and visualize the different stages of wing scale development. However, the self-assembly of block-copolymers can be seen as an analogue model for the development of wing scales [18],[42]. In block-copolymer systems perforated lamellar and gyroid structured phases are stable configurations lying close to each other in the phase diagram [43],[44]. It is hence very likely that in the developing cell that develops into the butterfly wing scale, the perforated multilayers and gyroid photonic crystals are formed due to slightly different development conditions. A change in the developmental conditions, similar to e.g. a change in the concentration resulting in a phase transition in polymers [43], may thus be enough to ‘switch’ between the development of a perforated multilayer or a gyroid in the scale lumen.

This is the first observation of such a close connection between the lamellar phase (perforated multilayers) and the gyroid phase (photonic crystals) between two closely related species in the same genus. Additional research into the wing scale development of the Cattlehearts might therefore give important physical and chemical insight into how these complex optical structures are formed, for instance by cell-mediated self-assembly.

### Wing pattern and mimicry

Many male Cattlehearts feature a similar wing patterning, and curiously, the colors of wing patches due to a multilayer or a gyroid photonic crystal in the scale lumen are nearly indistinguishable. This suggests a function of these wing patterns in mimicry rings and/or aposematic signaling, especially since many (strongly colored) Cattlehearts, like *P. sesostris*, are shunned by birds, their main predators [9].

## Conclusions

We conclude that our experimental observations on the pigmentation and photonic structures in the wing scales of the Cattlehearts add important insight into the evolution of butterfly wing scales, especially given the simultaneous observation of perforated multilayers and gyroids in wing scales of butterflies within the same genus. This shows that in addition to advanced phylogenetic tools based on DNA barcoding [45], the pigmentation and morphological parameters of butterfly wing scales provide an important instrument for a detailed analysis of phylogenetic trees. Especially the highly unlikely appearance of multiple traits in closely related butterflies (Figure 6A) should be taken into account when assembling maximum likelihood phylogenetic trees and incurring on the evolution of butterflies and specifically butterfly wing traits [34],[46].

## Methods

### Animals

Specimens of the butterflies were obtained commercially trough Tropical Butterflies and Insects of America (Tampa, FL, USA) and The Bug Maniac (Makassar, Indonesia). We furthermore investigated *Parides* butterflies in the Lepidoptera collection of the Natural History Museum Naturalis (Leiden, the Netherlands). Additional file 2: Table S1 summarizes the investigated specimens.

### Photography

The butterflies were photographed with a Canon EOS 550D camera (Canon Inc., Tokyo, Japan) equipped with a Canon 50 mm-macro objective and a ring flash. Details of the scale arrangement on the wings were photographed with a Zeiss Universalmikroskop (Carl Zeiss AG, Oberkochen, Germany) equipped with a Kappa DX-40 digital camera (Kappa GmbH, Gleichen, Germany).

### Spectrophotometry

Reflectance spectra of intact wings were measured with a bifurcated probe (Avantes FCR-7UV200, Avantes, Eerbeek, The Netherlands). The probe delivered light about normally to the wing plane and the also about normally reflected light was channeled to a photodiode array spectrometer (AvaSpec-2048-2, Avantes). The light source was a Deuterium-Halogen lamp (Avantes D(H)-S). A white diffuse reference tile (Avantes WS-2) served as a reference in all reflectance measurements. Transmittance spectra obtained from isolated wing scales, immersed in a fluid with refractive index 1.55 (Cargille Labs, Cedar Grove, NJ, USA), were measured with a microspectrophotometer (MSP), being a Leitz-Ortholux microscope, equipped with the Avantes light source and spectrometer. The measured transmittance, *T*(*λ*), yielded the absorbance (or optical density) *D*(*λ*) = − log_10_*T*(*λ*), where *λ* is the wavelength.

The angular distribution of light scattered by intact wings was measured with an angle-dependent reflectance measurement (ARM) setup, which consists of two optical fibers, mounted at two independent rotatable, but coaxial goniometers. A focused Xenon light source delivered light via one fiber to an area with diameter ~4 mm. The other fiber collected the reflected and scattered light from a wing area with diameter ~9 mm and delivered it to the Avantes spectrometer. A UV/VIS-polarizer was mounted in front of the detection fiber (see also [31],[41]). The wings were placed on a black cardboard and positioned at the collinear axes of rotation of the two goniometers. The white diffuse reference tile served as a reference.

### Imaging scatterometry (ISM)

The (far-field) spatial scattering pattern of single wing scales, glued to the end of a pulled glass micropipette, was investigated with an imaging scatterometer (ISM; [29],[31],[47]). The ISM is built around an ellipsoidal mirror, collecting light from a full hemisphere. The investigated objects, i.e. the wing scales, positioned in the mirror’s first focal point, were illuminated either with a narrow-aperture primary beam via a small hole in the ellipsoidal mirror or with a large-aperture secondary beam via a half-mirror. The far-field scattering pattern of the light backscattered by the sample was collected by a digital camera. Angle-dependent reflectance spectra were collected via a half-mirror using the Avantes spectrometer [31],[47]. A small piece of magnesium oxide served as a white diffuse reference object.

### Scanning electron microscopy (SEM)

The structure of the wing scales was investigated with a Philips XL30-ESEM scanning electron microscope (SEM). To prevent electric charging, the samples were sputtered with gold or palladium prior to imaging.

### Multilayer modeling

The reflectance of the wing scales was modeled by applying multilayer theory [30]. The ridges responsible for the scattering of light consist of alternating air (a) and cuticle (c) layers, with thickness *d*_a_ and *d*_c_ and refractive index *n*_a_ and *n*_c_. Although the cuticle refractive index is slightly dispersive [48], we have assumed for all wavelengths *n*_a_ =1.0 and *n*_*c*_ = 1.56, to simplify the analysis. The propagation of a light beam through the multilayer can be found from Snell’s law: *n*_a_ sin *θ*_a_ = *n*_c_ sin *θ*_c_, where *θ*_a_ and *θ*_c_ are the angles of incidence in air and cuticular medium. The wavelength of peak reflectance of a multilayer is given by the interference condition *λ*_max_ = 2(*n*_a_*d*_a_ cos *θ*_*a*_ + *n*_c_*d*_c_ cos *θ*_c_) [49],[50].

### Taxonomy

We have used the species-level taxonomy provided by Condamine et al. ([4]; slightly updated from [7]), where 17 species have been examined on the species-level by DNA barcoding, and the taxonomy provided by Möhn et al. [33], where 36 species have been grouped (for more details, see Additional file 1).

### Data accessibility

The data set supporting the results of this article is included within the article and its additional files.

## Competing interests

The authors declare that they have no competing interests.

## Authors’ contributions

BDW conceived the design of the study, BDW and NY carried out the experiments, DGS participated in the design and coordination of the study, BDW and DGS drafted the manuscript. All authors read and approved the final manuscript.

## Additional files

## Supplementary Material

Additional file 1: Figures S1 and S2 and additional details on the taxonomy. Click here for file

Additional file 2: Table S1. Investigated Cattlehearts, morphological parameters and pigments found in the wing scales. Click here for file
