# Supplementary material for: Pigmentary and photonic coloration mechanisms reveal taxonomic relationships of the Cattlehearts (Lepidoptera: Papilionidae: Parides)
Source: BMC Evol Biol. 2014 Jul 27;14:160. doi: 10.1186/s12862-014-0160-9 (PMC4236566; doi:10.1186/s12862-014-0160-9)
Supplement: Additional file 1: — Figures S1 and S2 and additional details on the taxonomy. [file s12862-014-0160-9-S1.docx]

Additional file 1

for

**Pigmentary and photonic coloration mechanisms reveal taxonomic relationships of the Cattlehearts (Lepidoptera: Papilionidae: *Parides*)**

by Bodo D. Wilts, N. IJbema and D.G. Stavenga

# Supplementary Figures


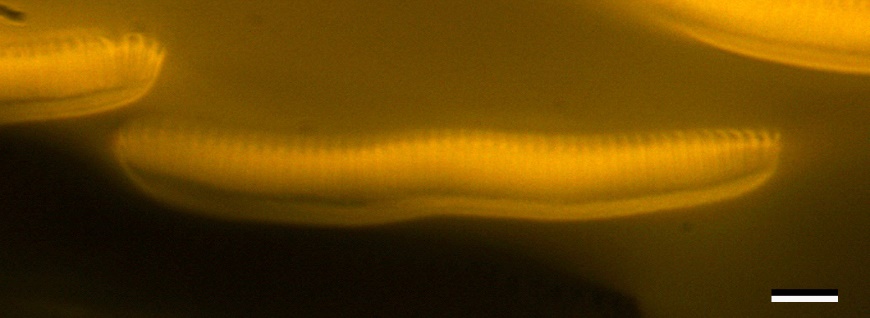


**Figure S1.** Fluorescence microscopy image of perpendicularly sectioned green wing scales of *P. aeanas*, using blue excitation light (450–490 nm) and a >520 nm barrier filter, showing that the scale’s pigment is mostly concentrated in the upper lamina. A SEM image of a cross-sectioned scale is shown in figure 1L; scale bar: 10 µm.

**Figure S2.** Reflectance spectra of the underside of green scales of *Parides aeneas*. The red curve is the average of five reflectance spectra measured with a microspectrophotometer; the dashed grey lines indicate the standard deviations. The blue curve is the reflectance spectrum modeled using classical multilayer theory.

# Taxonomy

We used different phylogenies which are hereafter classified in more detail.

## **Classification 1: Condamine et al. (2012) (refined results from Silva-Brandao et al. 2005)**

Technique: inferred from DNA barcoding with maximum parsimony, maximum likelihood (ML) and Bayesian inference (see also ref. Condamine et al., 2012; Silva-Brandao et al., 2005).

Number of included *Parides* species: 18

Silva-Brandao et al. (2005) divided *Parides* into four major lineages

- **G1**: *P.* *ascanius*, *P.* *bunichus*
- **G2**: *P.* *agavus*, *P.* *proneus*
- **G3**: *P.* *chabrias*, *P.* *childrenae*, *P.* *photinus*, *P.* *sesostris*, *P.* *anchises*, *P.* *vertumnus*
- **G4**: *P.* *aeneas*, *P.* *tros*, *P.* *eurimedes*, *P.* *neophilus*, *P.* *zacynthus*, *P.* *lysander*, *P.* *panthonus*

The species-level phylogenetic tree that was presented by Silva-Brandao et al. (2005) and was refined by Condamine et al. (2009) is shown in Figure 6A.

## **Classification 2: Möhn et al. (Möhn et al., 2007)**

Technique: unknown

Number of included *Parides* species: 36.

Möhn et al. (2007) divided the Cattlehearts, *Parides*, into 6 species groups (Fig. 6B):

- **Ascanius**: *P.* *agavus*, *P.* *alopius,* *P.* *ascanius,* *P.* *bunichus*, *P.* *gundlachianus*, *P.* *montezuma,* *P.* *phalaecus,* *P.* *photinus,* *P.* *proneus*
- **Klagesi**: *P.* *klagesi*
- **Chabrias**: *P.* *chabrias,*  *P.* *hahneli,* *P.* *mithras, P.* *pizarro, P.* *quadratus,* *P.* *vercingetorix*
- **Aeneas**: *P.* *aeneas*, *P.* *aglaope*, *P.* *burchellanus*, *P.* *echemon*, *P.* *eurimedes*, *P.* *lysander*, *P.* *neophilus*, *P.* *orellana*, *P.* *panthonus*, *P.* *tros*, *P.* *zacynthus*
- **Sesostris:** *P.* *childrenae*, *P.* *sesostris*
- **Anchises**: *P.* *anchises*, *P.* *cutorina*, *P.* *erithalion*, *P.* *iphidamas*, *P.* *panares*, *P.* *phosphorus*, *P.* *vertumnus*
